# Supplementary material for: Splice-Junction-Based Mapping of Alternative Isoforms in the Human Proteome
Source: Cell Rep. Author manuscript; Available in PMC 2020 Jan 15. (PMC6961840; doi:10.1016/j.celrep.2019.11.026)
Supplement: 2 [file NIHMS1546469-supplement-2.zip › DF1/PXD006675/RightAtrium/RightAtrium_30_NRAP_ASGDIASDLR.pdf]

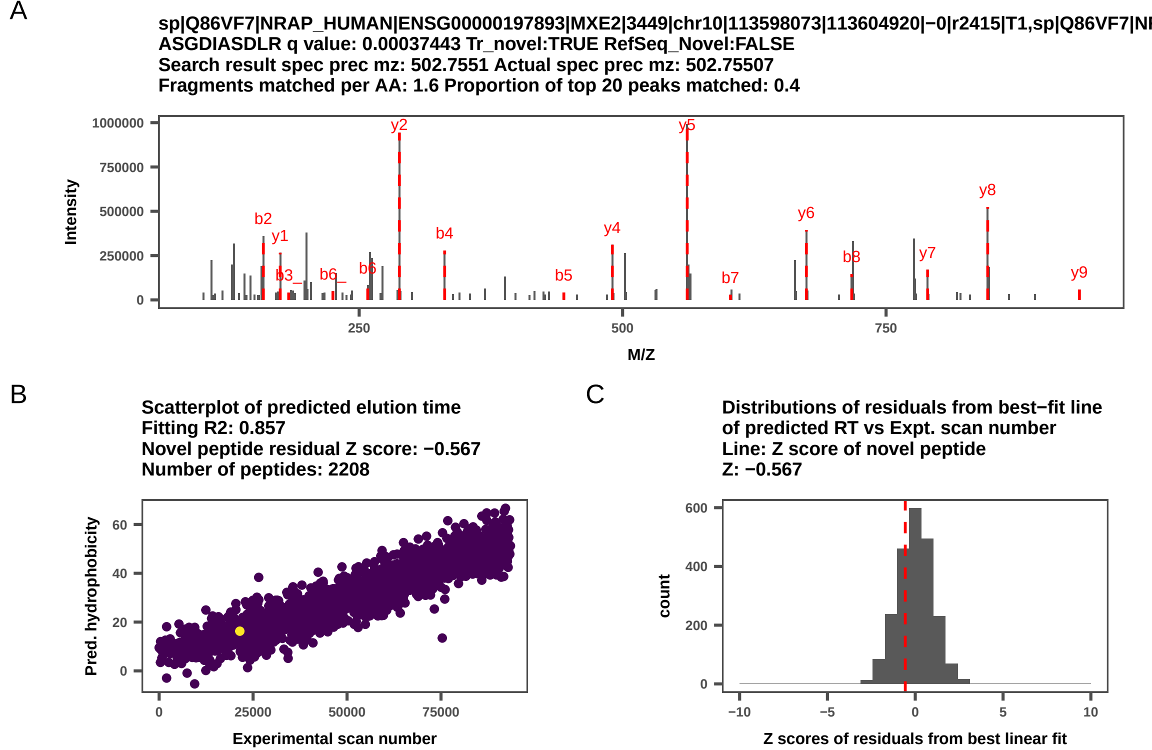
A
sp|Q86VF7|NRAP_HUMAN|ENSG00000197893|MXE2|3449|chr10|113598073|113604920|−0|r2415|T1,sp|Q86VF7|NI
ASGDIASDLR q value: 0.00037443 Tr_novel:TRUE RefSeq_Novel:FALSE
Search result spec prec mz: 502.7551 Actual spec prec mz: 502.75507
Fragments matched per AA: 1.6 Proportion of top 20 peaks matched: 0.4
Intensity
M/Z
y2
y5
y8
y6
y4
b2
y1
b4
b3_
b6_
b6
b8
y7
b5
b7
y9
1000000
750000
500000
250000
0
250
500
750
B
Scatterplot of predicted elution time
Fitting R2: 0.857
Novel peptide residual Z score: −0.567
Number of peptides: 2208
Pred. hydrophobicity
Experimental scan number
60
40
20
0
0
25000
50000
75000
C
Distributions of residuals from best−fit line
of predicted RT vs Expt. scan number
Line: Z score of novel peptide
Z: −0.567
count
Z scores of residuals from best linear fit
600
400
200
0
−10
−5
0
5
10
